# Biological Safety and Biodistribution of Chitosan Nanoparticles

**DOI:** 10.3390/nano10040810

**Published:** 2020-04-23

**Authors:** Dmitry Sonin, Evgeniia Pochkaeva, Sergei Zhuravskii, Viktor Postnov, Dmitry Korolev, Lyubov Vasina, Daria Kostina, Daria Mukhametdinova, Irina Zelinskaya, Yury Skorik, Elena Naumysheva, Anna Malashicheva, Pavel Somov, Maria Istomina, Natalia Rubanova, Ilia Aleksandrov, Marina Vasyutina, Michael Galagudza

**Affiliations:** 1 Institute of Experimental Medicine, Almazov National Medical Research Centre, 2 Akkuratova Street, 197341 Saint Petersburg, Russia; zhuravskiy_sg@almazovcentre.ru (S.Z.); postnovvn@mail.ru (V.P.); korolev_dv@almazovcentre.ru (D.K.); lubov.vasina@gmail.com (L.V.); kostina_da@almazovcentre.ru (D.K.); mukh.dv@yandex.ru (D.M.); zelinskaya_ia@almazovcentre.ru (I.Z.); yury_skorik@mail.ru (Y.S.); helinchik@yandex.ru (E.N.); malashicheva_ab@almazovcentre.ru (A.M.); istomina_ms@almazovcentre.ru (M.I.); rubanova_ns@almazovcentre.ru (N.R.); aleksandrov_iv@almazovcentre.ru (I.A.); vasyutina_ml@almazovcentre.ru (M.V.); 2 Laboratory of Biophysics of Blood Circulation, Pavlov First Saint Petersburg State Medical University, 6–8 L’va Tolstogo Street, 197022 Saint Petersburg, Russia; 3 Graduate School of Biotechnology and Food Science, Peter the Great Saint Petersburg Polytechnic University, 29 Polytechnicheskaya Street, 195251 Saint Petersburg, Russia; pochkaeva_ei@spbstu.ru; 4 Chemical Faculty, Saint Petersburg State University, 13B Universitetskaya Embankment, 199034 Saint Petersburg, Russia; 5 Laboratory of Regenerative Biomedicine, Institute of Cytology, Russian Academy of Sciences, 4 Tikhoretsky Avenue, 194064 Saint Petersburg, Russia; 6 Laboratory of Natural Polymers, Institute of Macromolecular Compounds, Russian Academy of Sciences, 31 Bolshoy Avenue V.O., 199004 Saint Petersburg, Russia; 7 TESCAN (CIS) Ltd., 11 Grazhdansky Avenue, 195220 Saint Petersburg, Russia; pavel.somov@tescan.ru; 8 Department: Micro- and Nanotechnology, Saint Petersburg Electrotechnical University “LETI”, 5 Professora Popova Street, 197376 Saint Petersburg, Russia; 9 Laboratory of Digital and Display Holography, ITMO University, 49 Kronverksky Avenue, 197101 Saint Petersburg, Russia

**Keywords:** chitosan, polymer nanoparticles, biodistribution, blood compatibility, safety evaluation, in vivo treatment

## Abstract

The effect of unmodified chitosan nanoparticles with a size of ~100 nm and a weakly positive charge on blood coagulation, metabolic activity of cultured cardiomyocytes, general toxicity, biodistribution, and reactive changes in rat organs in response to their single intravenous administration at doses of 1, 2, and 4 mg/kg was studied. Chitosan nanoparticles (CNPs) have a small cytotoxic effect and have a weak antiplatelet and anticoagulant effect. Intravenous administration of CNPs does not cause significant hemodynamic changes, and 30 min after the CNPs administration, they mainly accumulate in the liver and lungs, without causing hemolysis and leukocytosis. The toxicity of chitosan nanoparticles was manifested in a dose-dependent short-term delay in weight gain with subsequent recovery, while in the 2-week observation period no signs of pain and distress were observed in rats. Granulomas found in the lungs and liver indicate slow biodegradation of chitosan nanoparticles. In general, the obtained results indicate a good tolerance of intravenous administration of an unmodified chitosan suspension in the studied dose range.

## 1. Introduction

Chitosan, the product of the complete or partial deacetylation of chitin, is a widely used excipient for topical or oral drug administration [1,2,3,4,5]. It is also now viewed as a useful material for the construction of drug and gene delivery systems for parenteral administration [1,6,7,8,9,10,11]. The prospect of using chitosan as a carrier matrix for drug delivery emphasizes the importance of knowing the biological safety of chitosan dispersal systems. This is especially the case when chitosan is supplied as a nanoscale formulation, as this raises issues about nanotoxicity.

The potentially toxic attributes of nanomaterials are associated with the size, shape, and electrokinetic potential (ζ-potential) of the particles and less dependent on the chemical composition of the matrix and its biodegradation products [12,13,14,15,16]. Disperse nanomaterial systems are now considered generally safe if applied as nanoparticles that meet a size criterion in the range of 150–300 nm [15,17]. Particles larger than 300 nm can be eliminated from the human body by resident macrophages [13,15], but the activation of macrophages triggers aseptic inflammation, often with a granulomatous component (e.g., in the lungs and liver) [18,19,20].

The charge of the nanoparticle can also create biocompatibility issues. For chitosan nanoparticles (CNPs), charge biocompatibility is achieved by regulating the ζ-potential. Chitosan is a cationic polymer (pKa about 6.5) that carries a positive charge under physiological conditions. CNPs with a large positive ζ-potential (about 50 mV) cause hemolysis [21,22] and thrombosis [23] when they come into contact with blood. Conversely, chitosan nanoparticles modified to have a large negative ζ-potential (−40 mV) activate macrophages and phagocytosed by them [13], processes that again lead to aseptic inflammation [21,24]. The current literature indicates that the hemocompatibility criterion can be met by nanoparticles that have mean values for the positive and negative ζ-potential ranging from +15 to −30 mV [21,25,26]. For these reasons, biocompatibility is usually achieved with nanoparticles with sizes ranging from 150 to 300 nm and ζ-potential ranging from +15 to −30 mV.

Most studies that have addressed the issue of nanotoxicity of CNPs and chitosan-based nanoparticles have usually performed the biocompatibility studies 2–4 days after intravenous administration in animal models [25,27,28,29]. However, the toxicities determined by these types of studies, which use short periods of observation for long-circulating dispersed solutions [26,28], may not be fully representative since neither the biodistribution nor the biodegradation processes are completed during these short periods. Nevertheless, studies with longer periods of observation after intravenous administration in subacute or chronic experiments are rare [30], even though long-term observations of the biological effects of dispersed systems are no less important than the analysis of acute toxicity. These tasks require complex experimental work, including the study of organ biodistribution, a systemic response to the administered drug, reactive changes in the tissues of target organs, and the biodegradation intensity of already internalized polymer matrices.

Chitosan nanoparticles are considered as suitable systems for delivering cardioprotective drugs to treat myocardial ischemia reperfusion injury [7,31]. Intravenously injected chitosan nanoparticles do not accumulate in the intact myocardium regardless of the ζ-potential [13], herewith Hwang et al. (2014) published evidence of retention of chitosan hydrogel nanoparticles in injured myocardium in vivo. In this regard, we are particularly interested in the cardiotoxicity of chitosan nanoparticles [31].

The aim of the present study was to determine the acute and subacute toxic effects of intravenous administration of chitosan nanoparticles (CNPs) over a 14-day period.

## 2. Materials and Methods

### 2.1. Characterization of Chitosan Sample

Low molecular weight chitosan from crab shells (MW 50–190 kDa, degree of deacetylation 75%–85%; Sigma-Aldrich Corp. St. Louis, MO, USA) was used in this study. The viscosity average molecular weight (M_η_ = 138 kDa) was calculated using the Mark–Houwink equation [η] = 3.41 × 10^–3^ × M_η_^1.02^ [32]. The intrinsic viscosity of chitosan ([η] = 5.94 dL/g) was determined using an Ubbelohde capillary viscometer (Design Bureau Pushchino, Pushchino, Russia) at 20 °C with 0.33 M acetic acid and 0.3 M NaCl as solvent.

The degree of deacetylation (DDA = 84 ± 2%) was determined using ^1^H NMR spectroscopy. Samples for NMR spectroscopy were prepared by dissolving 5 mg chitosan in 0.5 mL D_2_O + 5 µL CF_3_COOH. The spectra were recorded using Bruker Avance 400 spectrometer (Bruker, Billerica, MA, USA) at 70 °C using a zgpr pulse sequence with suppression of residual H_2_O.

### 2.2. Preparation of Chitosan Nanoparticles

The CNPs were obtained by grinding chitosan sample in a PM 100 CM planetary ball mill (Retsch GMBH, Haan, Germany) in a 125 mL grinding jar containing 5 mm diameter steel balls. A 5 g sample of chitosan and 50 mL of distilled water were placed in the grinding jar and processed for 1 h at 250 rpm. After grinding, the solution containing the CNPs was filtered through an Omnipore membrane filter (Merck Millipore, Darmstadt, Germany; 0.2 μm pore size). The concentration of chitosan nanoparticles in the stock suspension was 0.5 mg/mL, which was the maximum achieved concentration of CNP and stable for a week.

**Figure 1:**
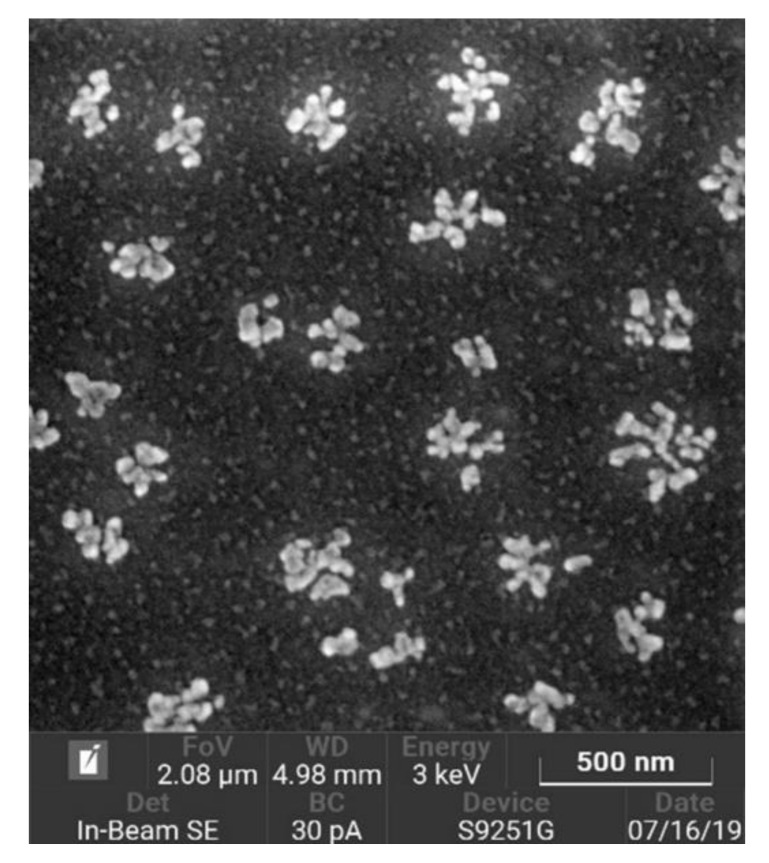
Scanning electron microscopy (SEM) image of chitosan nanoparticles. Clusters of nanoparticles are not aggregates but individual particles that have come together during drying.

The CNP size was estimated by dynamic light scattering using a Malvern Zetasizer 3000 (Malvern Instruments Ltd, Malvern, Worcestershire, U.K.) and non-invasive backscatter technology. The value of the ζ-potential in water was measured on the same device using the Smoluchowski equation. The results indicated that particles with a diameter of 100 ± 30 nm and ζ-potential of +10 ± 1 mV had been obtained. The particle shape was evaluated using a Tescan S9251G scanning electron microscope (Tescan, Brno, Czech Republic) at an accelerating voltage of 3 kV with an in-beam secondary electron detector (Tescan, Brno, Czech Republic) (Figure 1).

### 2.3. In Vitro Experiments

#### 2.3.1. Assessment of Hemolytic Activity

The ability of CNP particles to cause acute hemolysis was evaluated using venous blood taken from rats 1 h after the start of the experiment (see “2.4.1.1. Assessment of CNP Effect on Hemodynamic Parameters”). The blood was collected from the posterior vena cava using a syringe containing a blood stabilizer (heparin, 10 U/cm^3^). The blood was centrifuged prior to the analysis, to remove intact red blood cells. The free hemoglobin content in plasma was measured photometrically using a photoelectric colorimeter KFK-2MP (JSC “Zagorsk Optical and Mechanical Plant”, Sergiev Posad, Russia) at a wavelength of 540 nm in glass cuvettes with a 10 mm path length, as described previously [33]. Positive control experiments were performed to determine the optical density of solutions obtained on the basis of artificially hemolyzed 5 ± 2% red blood cell mass by mixing with distilled water in proportion 1:2. The data is presented as the mean absorbance ± SEM.

#### 2.3.2. Assessment of Anticoagulant Activity

The anticoagulant properties of a dispersed CNP solution were evaluated by its ability to lengthen the clotting time (compared to controls) when added to platelet-poor plasma. Measurements included the activated partial thromboplastin time (APTT), prothrombin time (PT), and thrombin time (TT) [34].

Human blood for the study was taken from healthy volunteers (*n* = 5) obtained at the Donor Center (blood transfusion station) of both sexes, aged 25–40 years, who had not taken any drugs that could affect platelet function for the previous 7–10 days. Platelet activation was prevented by collecting the blood in Apexlab vacuum tubes (Yancheng Huida Medical Instruments Co., Ltd., Yancheng, China) containing 3.8% sodium citrate (0.129 M) as a stabilizer at a ratio of 1:9 to whole blood.

The CNP dispersions in a volume of 0.5 mL at final chitosan concentrations of 0.25%, 0.125%, and 0.06% were added to 0.5 mL of prepared blood. Clotting times were evaluated with an automated hemostasis analyzer (STA Compact) using reagent kits for determining activated partial thromboplastin time (STA APTT), prothrombin time (STA Neoplastin Plus), and thrombin time (STA Thrombin) (Roche Diagnostics, Basel, Switzerland).

#### 2.3.3. Assessment of Antiplatelet Activity

The aggregation potential of the obtained CNPs was studied with the ADP-induced platelet aggregation test [33]. The effect of CNPs on induced platelet aggregation was determined at a final chitosan concentration of 0.25%, 0.125%, and 0.06% using a whole blood impedance aggregometer (4-channel) (Model 590, Chrono-log Corporation, Havertown, PA, USA) with the Chrono-par ADP reagent (Chrono-log Corporation, Havertown, PA, USA) [35].

#### 2.3.4. Study of Cytotoxic Properties

The MTT assay was performed by the cultivation method described by Niks and Otto [36]. The following concentrations of CNP suspensions in saline were tested (final concentrations in the wells): 0.0005%, 0.005%, and 0.05%. The optical density of cells stained with MTT was measured using a Bio-Rad spectrophotometer (Bio-Rad, Hercules, CA, USA) at 550 nm. Inhibition of the vital activity of cultured HL-1 cardiomyocytes was monitored by a decrease in optical density. All studies were performed in triplicate.

### 2.4. In Vivo Experiments

These studies were performed on 24 male SPF Wistar rats weighing 225–250 g and housed under barrier-type vivarium conditions in individually ventilated cells. Rats were maintained under a 12-h light-dark regime and were offered a standard diet and water ad libitum. All procedures with animals (obtaining blood samples, introducing the dispersed CNP solutions into the femoral vein) were performed under general anesthesia with 2–5% isoflurane in the SomnoSuite anesthesia system (Kent Scientific, Torrington, CT, USA).

The experiments were performed in compliance with the principles of human treatment of animals, regulated by the requirements of the European Convention (Strasbourg, 1986) for the housing, feeding, and care of experimental animals and in accordance with the “Guide for the Care and Use of Laboratory Animals” (National Institute of Health publication No. 85-23, USA) and “Guideline for experimental (preclinical) studying of new pharmacological substances.” All procedures involving animal use in the study were reviewed and approved by the Commission for Control of the Housing and Use of Laboratory Animals of the Almazov National Medical Research Centre and were deemed in compliance with the ethical requirements for the handling of laboratory animals.

#### 2.4.1. Biosafety Study of Single Intravenous CNP Administration

All animals were randomized into two groups. Rats in the control group received saline (0.9% NaCl) in a volume of 8 mL/kg for 10 min (*n* = 6). Rats in the experimental group received intravenous administration of a CNP suspension in saline at the following doses: 1st subgroup (CNP-1)—1 mg/kg; 2nd subgroup (CNP-2)—2 mg/kg; and 3rd subgroup—4 mg/kg (CNP-4). Each group contained six rats.

##### Assessment of CNP Effect on Hemodynamic Parameters

This study was performed according to the methods described previously [37]. A suspension of nanoparticles in saline at a chitosan concentration of 0.05% was administered intravenously to anesthetized rats through a catheter inserted into the femoral vein. The dosage was 2 mg/kg of CNP for 10 min in a volume of 4 mL/kg. Experiments were performed under conditions of spontaneous breathing of the animals. Hemodynamic parameters were observed and monitored for 30 min before and 60 min after administration of the test substances.

##### Assessment of General Toxicity

The general toxicity effect of CNP administration was evaluated by monitoring the dynamics of body weight: at initiation of the experiment and then at 1 day, 7 days, and 2 weeks after the administration of the studied substances.

##### Hematological Analysis

Blood samples for hematological analysis were taken from the retro-orbital sinus at three timepoints: immediately before administration, 24 h after administration, and after 14 days (at euthanasia). BD Vacutainer tubes pre-coated with K3-EDTA were used for blood collection.

The following hematological parameters were evaluated: leukocytes (WBC, ×10^9^/L), leukocyte fractions: granulocytes (Gr, %), lymphocytes (Lym, %), monocytes (Mi, %), erythrocytes (RBC, ×10^12^/L), and hemoglobin (Hb, g/L). The erythrocyte indices included: mean corpuscular hemoglobin (MCH, pg), mean corpuscular hemoglobin concentration (MCHC, g/L), mean corpuscular volume (MCV, fl), and hematocrit (Hct, %). A veterinary hematological analyzer Abacus Junior Vet (Diatron, Budapest, Hungary) was used for these analyses.

##### Blood Chemistry Analysis

Blood biochemical parameters were evaluated once, at euthanasia. The alanine aminotransferase (ALT), aspartate aminotransferase (AST), alkaline phosphatase, creatine phosphokinase-MB (CPK-MB), creatinine, lactate dehydrogenase (LDH), bilirubin, total protein, and urea levels were determined using an automated chemistry analyzer (ChemWell Combo, Awareness Technology Inc., Palm City, FL, USA) and commercial reagent kits (Spinreact, Girona, Spain; Cloud-Clone Corp., Katy, TX, USA). Venous blood was taken from the inferior vena cava for these analyses.

#### 2.4.2. Study of CNP Biodistribution in Rats

The nature and dynamics of the CNP biodistribution in rats was studied at the macro level by a novel method using in vivo detection of particles labeled with indocyanine green (ICG) (ICG-CNPs).

##### Preparation of ICG-CNPs

Chitosan has a free amino group in each monomeric unit, and ICG, in turn, has a sulfonic acid group, and interaction between the protonated amine groups in chitosan and the sulfonate groups in ICG leads to a rather strong ionic bonding [38].

The synthesis of ICG-CNPs was performed as follows: 1 mL of an aqueous solution of ICG (0.5 mg/mL) was added to 2 mL of an aqueous CNP suspension. The resulting colloidal solution was mixed on a shaker (LS-220, Loip, Saint Petersburg, Russia) at a rate of 300 rpm for 2 h. The resulting product was washed five times by centrifugation at 3000 rpm for 5 min and decanting the supernatant. The resulting pellet was resuspended in 10 mL distilled water and dispersed for 1 min with an UZD-2 ultrasonic disperser (VNIITVCH, Saint Petersburg, Russia). After washing, the suspension was recentrifuged and the pellet was dispersed in 2 mL distilled water.

##### Assessment of ICG-CNP Biodistribution

The ICG-CNP biodistribution was studied on a Lumina III fluorescence tomography system (PerkinElmer, Waltham, MA, USA). The ICG-CNP fluorescence was monitored at 810 nm after excitation with light at 780 nm. Before the study, Zoletil-anesthetized animals were shaved on the ventral area. A bolus dose of ICG-CNPs (5.12 mg/kg) or ICG (0.028 mg/mL) in a volume of 8 mL/kg was then administered intravenously (*n* = 3 animals per group). The anesthetized animals were placed in the supine position in the chamber of the Lumina III, where the temperature was maintained at 37 °C. Fluorescence was monitored initially and then every 5 min for 30 min after administration of the test substances. After the last scan was completed, the major organs (heart, lungs, liver, spleen, and kidneys) were harvested to record the fluorescence from the surface of the organs.

##### Histological Examinations

Histological examinations were performed to determine the response of vital organs to the administration of the test suspensions. The animals were euthanized on the 14th day after CNP administration for collection of biomaterials for the study. The liver, lungs, kidneys, heart, and spleen were harvested for histological examination. The organs were weighed to obtain mass coefficients according to the formula: MC = organ mass (g)/body weight (g) × 100%.

Fragments from each organ were then fixed in 10% neutral formalin in phosphate buffer (pH 7.4), dehydrated in a graded ethanol series, and embedded in paraffin blocks according to standard histological techniques. Paraffin sections, 5 μm thick, were stained with hematoxylin and eosin for histological evaluation.

The state of the liver and lung tissues were also assessed by morphometric evaluation. The numbers of Kupffer cells and granulomas, and their sizes were determined from digital images of liver preparations. The width of the alveolar septa (μm), number of interstitial macrophages per unit area, and number and area of granulomas were evaluated on digital images of lung preparations. The mean area of granulomas was calculated in 10 randomly selected foci of infiltrates on one section from each animal using the function of manual selection of borders and the subsequent automatic calculation of the area with a 40× objective lens and a 10× eyepiece lens.

##### Morphological Visualization of CNP Internalization

A study of the nature of internalization of chitosan-containing particles was performed after a single intravenous administration of a CNP suspension in saline at a dose of 16 mg/kg in a 2.0 mL volume (*n* = 4 animals). The histochemical reaction described by Grocott (Biovitrum LLC, Saint Petersburg, Russia) was used for this task. The rats were euthanized 2 h after injection of the CNP suspension. The biodistribution of Ag-positive inclusions in the studied organs (liver, lungs) was evaluated visually using a light microscope.

##### Immunohistochemical Analysis

Immunohistochemistry (IHC) analysis was used to clarify the cellular composition of granulomas found in the lungs and liver. A primary monoclonal mouse antibody (Anti-CD68 antibody, ab 31630; Abcam, Cambridge, U.K.) was used at a dilution of 1:1000 at room temperature and a 1 h exposure. A biotin-free multimer detection system (D&A, Reveal-Biotin-Free Polyvalent DAB, Spring Bioscience Corp., Pleasanton, CA, USA) was used to detect bound primary antibodies. The preparations were stained with Mayer’s hematoxylin (Bio-Optica, Milano, Italy).

The histological evaluations, microphotography, and morphometry were performed using a Nikon Eclipse Ni-U upright microscope (Nikon Corporation, Tokyo, Japan) with magnifications of 100×, 200× and 400×, a Nikon DS-Fi2 high-definition color camera (Nikon Corporation, Tokyo, Japan), and NIS-Elements BR (v. 4.3) imaging software (Nikon Corporation, Tokyo, Japan).

### 2.5. Statistical Analysis

The median and interquartile range and the 25th and 75th percentiles (Me [25%–75%]) were used to describe data not showing a normal distribution. The t-test (paired Wilcoxon test) was used to assess the significance of differences before and after administration. The Mann–Whitney U test was used to assess the significance of differences between two nonconjugated populations. Differences were considered significant at *p* < 0.05.

## 3. Results

### 3.1. In Vitro Assessment of CNP Hemocompatibility

#### 3.1.1. Hemolytic Activity

**Figure 2:**
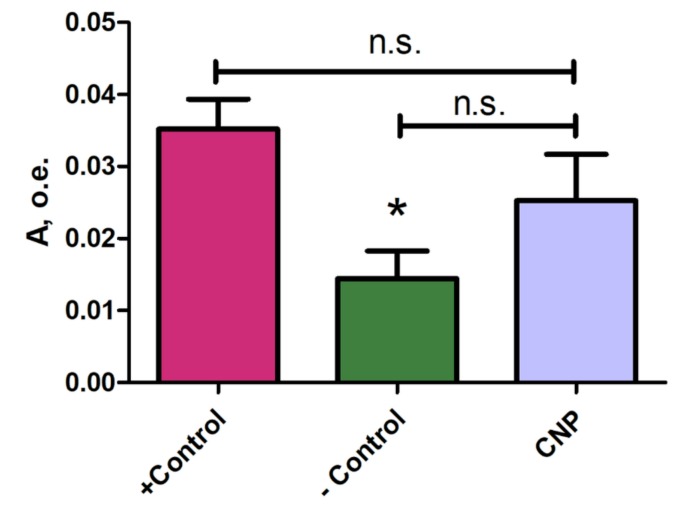
Absorbance (A) of rat plasma 60 min after intravenous administration of the suspension of chitosan nanoparticles (*n* = 7). +Control—positive control – artificially hemolyzed 5 ± 2% red blood cell suspension (*n* = 5); –Control—negative control (saline, *n* = 5). Mean ± SEM. * – *p* < 0.05 with respect to +Control. n.s.—nonsignificant. Increased plasma-free hemoglobin level is a sign of hemolysis. If intravenously administrated CNPs cause in vivo hemagglutination and hemolysis, then free hemoglobin should appear in the blood plasma samples, which is determined by the absorption values at a wavelength of 540 nm.

The absorbance (A) values of rat plasma samples one hour after intravenous administration of the CNP suspension were within the measurement error and did not show significant differences from the negative control group, indicating an absence or very low hemolysis during systemic administration of the CNP suspension at dose 4 mg/kg (Figure 2).

#### 3.1.2. Effect of CNP Suspension on Coagulation Hemostasis

A statistically significant decrease was observed for the prothrombin index (PI). A tendency to a decrease was seen for the APTT, and an increase was noted for the TT when a suspension of CNPs was mixed with human plasma (Table 1).

**Figure 3:**
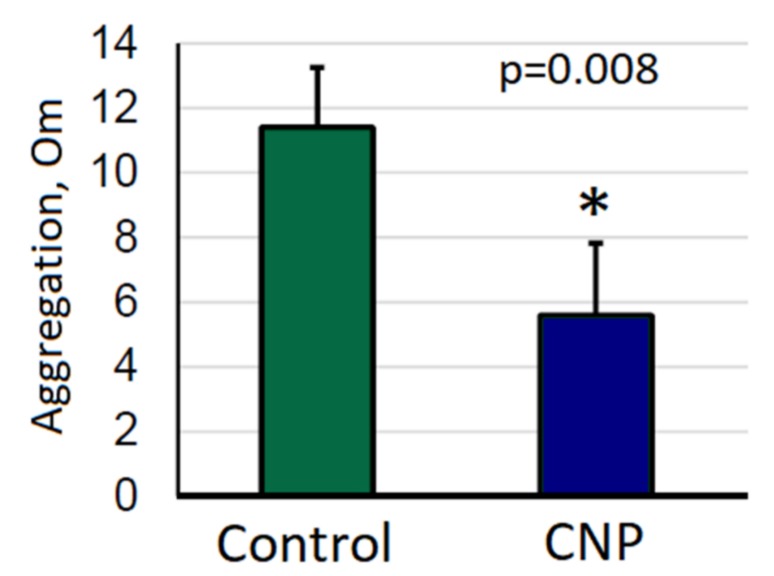
Platelet hemostasis in the ADP-induced platelet aggregation test with 0.12% CNP suspension added to human plasma. Mean ± standard error. In the ADP test, the CNPs significantly inhibited platelet aggregation in comparison with the control. * – *p* < 0.05 with respect to Control.

The data indicated a slight anticoagulant effect caused by the CNP suspension. A sample of chitosan-containing suspension showed a statistically significant antiplatelet activity in the ADP-induced platelet aggregation test (Figure 3).

#### 3.1.3. Cytotoxic CNP Properties

**Figure 4:**
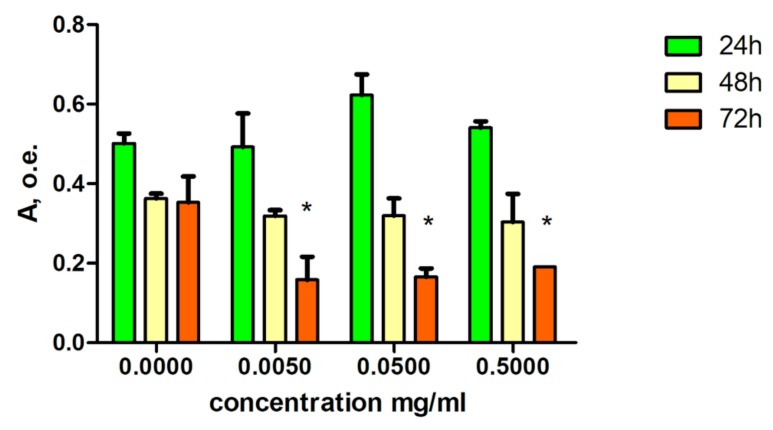
The effect of a suspension of chitosan nanoparticles on the viability of cultured cardiomyocytes. Cell viability was measured by MTT assay. Absorbance (A) positively correlates with number of alive cells. CNPs reduce cell viability in tested concentrations only after 72 h incubation. Mean ± SEM. * – *p* < 0.05 with respect to basal.

The metabolic activity of cultured cardiomyocytes in the presence of CNPs decreased by 10%–20% after 48 h. This decrease reached 50% of the control activity by the third day of cell incubation for all three CNP concentrations (Figure 4).

### 3.2. In Vivo Results of Acute Experiments

#### 3.2.1. Effect of CNP Suspension on Systemic Hemodynamics

The baseline levels of mean arterial pressure (MAP) and heart rate (HR) did not differ in the control and experimental groups (Table 2).

Administration of saline did not lead to significant changes in systemic hemodynamics. A short-term increase was noted in arterial pressure at 5 min after the end of administration of CNP suspension (2 mg/kg). Arterial pressure was restored to its baseline levels after 30 min. No statistically significant changes occurred in heart rate.

#### 3.2.2. In Vivo Biodistribution of CNPs

The corporal pattern of ICG fluorescence was identical at equal concentrations of ICG (0.028 mg/mL) in the injected solutions. One distinctive feature of the administration of free ICG was the appearance of ICG fluorescence in the intestinal projection at the 20 min timepoint of the experiment (not shown).

**Figure 5:**
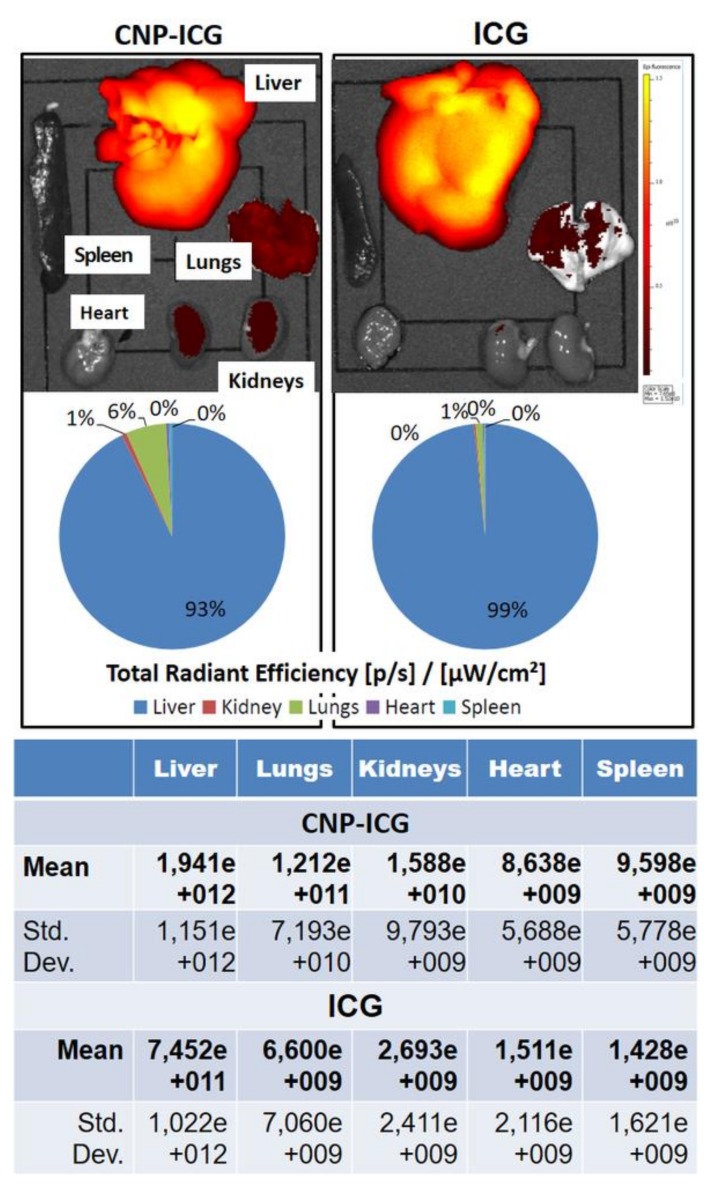
In loco biodistribution pattern of free and CNP-immobilized ICG 30 min after intravenous administration. The diagrams show the percentage of fluorophore accumulation in organs measured as total radiation efficiency ([p/s]/[µW/cm^2^]). Percentage are calculated from the mean of total radiant efficiency (given in the table) for each organ (*n* = 3).

As expected, the liver was the primary target of accumulation of both free and bound ICG, which was confirmed in images taken on the surface of the organs in near infrared light using fluorescence tomography (Figure 5). By contrast, the introduction of immobilized ICG led to accumulation in the lungs (6%) and kidneys (1%).

### 3.3. General Toxicity of CNP Suspension

**Figure 6:**
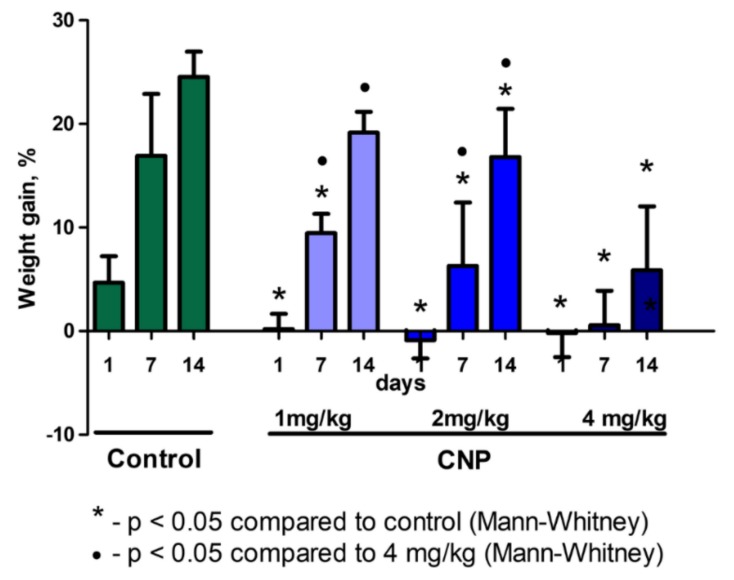
Dynamics of body weight after intravenous administration of CNPs (% of the baseline). The Mann–Whitney test was used to assess the significance of differences between groups. Data shown are median ± range.

A dose-dependent deprivation effect on body weight was observed in the experimental groups within 14 days. An inverse dependence of the increase in body weight on the dose of injected suspensions was also noted (Figure 6).

**Figure 7:**
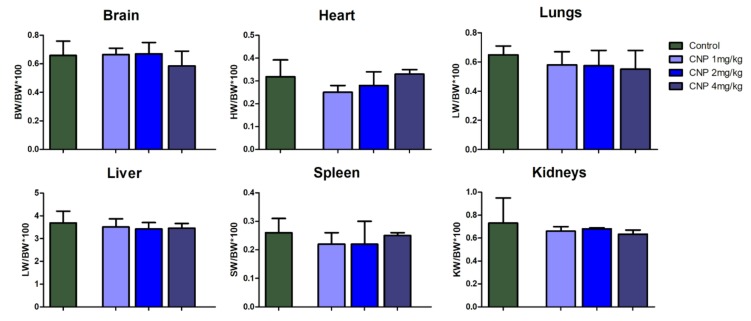
Mass ratios of rat organs 14 days after intravenous administration of CNP suspension. Median ± range. The ratio of organ mass to body weight is used in toxicology to detect target organs of the toxicant. There were no significant effects of CNPs on mean weight ratios of the organs.

Analysis of the mass ratios (MR) revealed no differences for any of the organs between the control and CNP subgroups. (Figure 7).

#### 3.3.1. Results of Hematological Analysis

**Figure 8:**
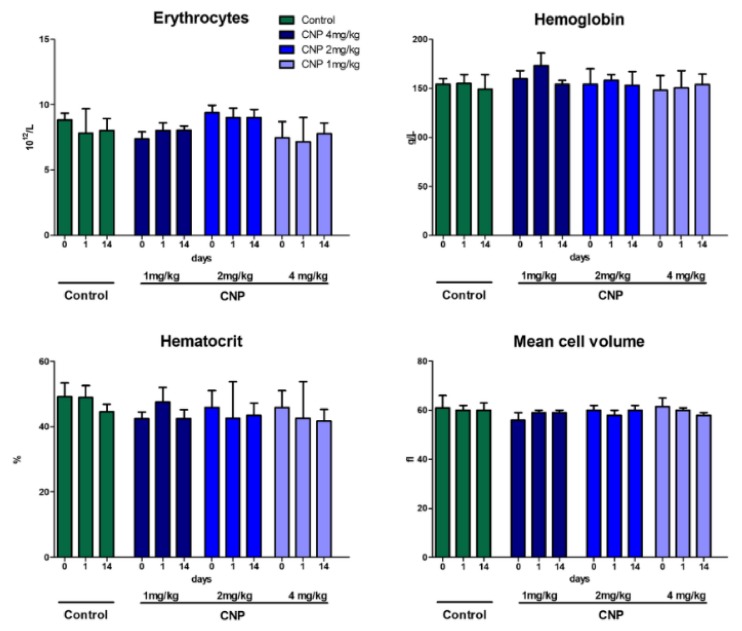
Dynamics of rat hemoglobin and erythrocyte parameters at different time points (0, 1 and 14 days) after intravenous injection of CNPs (1–4 mg/kg). Data represent the median ± range. These data complement results of the analysis of the level of free hemoglobin in the blood plasma of rats after administration of CNPs. If CNPs caused significant intravascular hemolysis and thrombosis, all red cell mass parameters (erythrocytes, hematocrit, and hemoglobin) can be decreased by CNPs–erythrocyte interactions, while MCV within normal range in the first day after intravenous administration of CNPs. The results of hematological analysis did not reveal significant changes in the parameters of red blood.

Hemoglobin levels, as well as the size and number of red blood cells, did not differ between the groups at the baseline (Figure 8). The CNP administration did not affect the hemoglobin content, size, or number of red blood cells.

**Figure 9:**
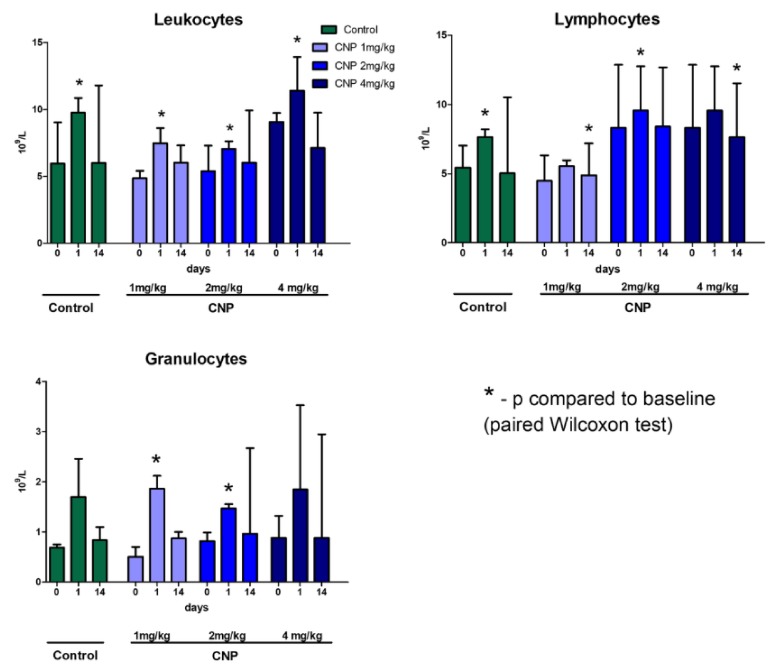
Dynamics of rat leukocyte parameters. Median ± range. Changes in white blood cells count were observed 1 day after injection, which were caused by minor surgical intervention (venesection). An increase in the level of white blood cells in the blood is a sign of a systemic immune response to tissue/organ injury or invasion. In this study, the dose of chitosan nanoparticles administered intravenously was not enough to cause a significant increase in white blood cell levels.

Baseline parameters of the leukocyte pools were similar between the experimental groups. An increase in the leukocyte blood levels was observed at 1 day after the administration in all groups (Figure 9), which reflected a reaction to the surgical trauma of the femoral vein catheterization. After 2 weeks, the leukocyte levels returned to normal.

#### 3.3.2. Results of Biochemical Blood Tests

**Figure 10:**
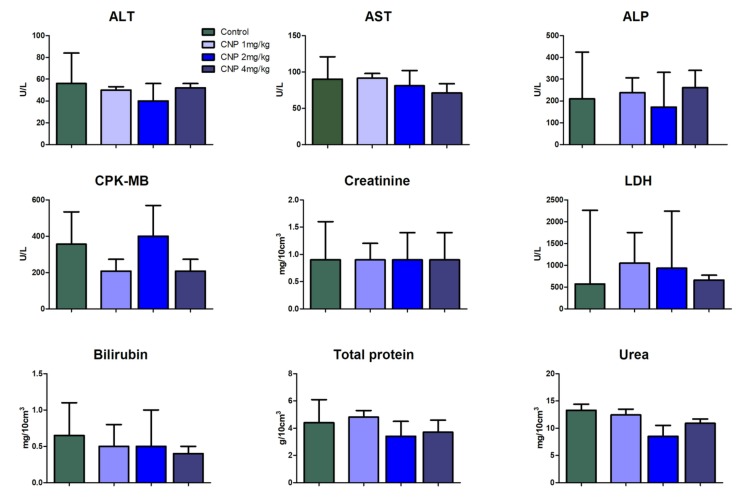
Rat blood biochemical parameters at 14 days after intravenous CNP administration. Median ± range. CNPs were mostly accumulated in the liver and in a lesser extent in the lungs. Kidneys, spleen, and heart accumulated about one percent of all nanoparticles. CNP could affect these organs and impair their functions. Biochemical parameters related to the liver, kidney, and heart did not reveal any signs of injury or malfunction.

Figure 10 shows the biochemical levels for rat blood at 14 days after the administration of CNPs. The measured biochemicals are markers of hepatic and myocardial cytolysis, intrahepatic obstruction of the bile ducts, inhibition of the biosynthetic liver functions, and disorders of glomerular filtration and reabsorption in the kidney tubules. None of these markers differed from the control levels for the same observation periods. Normal reference range of biochemical parameters of Wistar male rats: (a) related to the liver - aspartate aminotransferase 131.0 ± 44.0 U/L, alanine aminotransferase 76.1 ± 11.4 U/L, lactate dehydrogenase 435.0 ± 102.0 U/L, alkaline phosphatase 237.1 ± 33.9 U/L, total bilirubin 1.7 ± 0.1, total protein 6.9 ± 0.6 g/dL; (b) related to the heart, creatine kinase MB 494.0 ± 109.0 U/L; (c) related to the kidney, creatinine 0.49 ± 0.01 mg/dL, urea 12.7 ± 1.8. (https://www.envigo.com).

#### 3.3.3. Results of Histological Analysis

##### Liver

**Figure 11:**
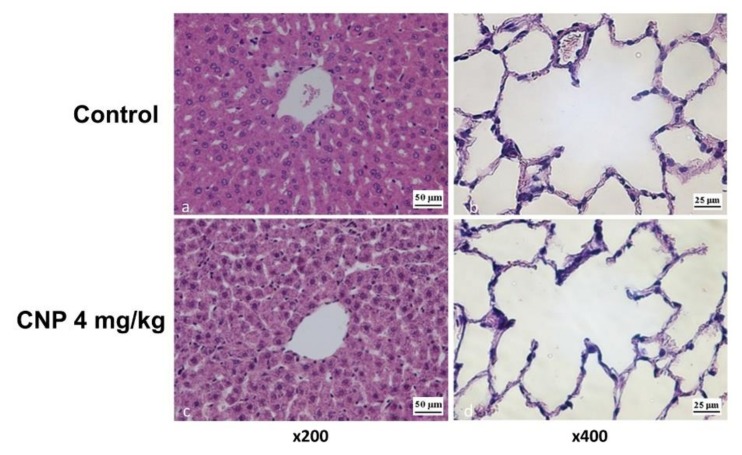
The histological pattern of rat organs at 14 days after a single injection of a CNP-4 suspension (4 mg/kg). Tissues were stained with hematoxylin/eosin.

Administration of CNPs at doses of 1–4 mg/kg for 14 days caused no alterations in the architectonics of the liver lobules. The number of Kupffer cells decreased at dosage of 4 mg/kg but was not significantly different from the number in the control group (37 ± 9 vs. 32 ± 7/0.141 mm^2^). Round cell granulomas with an average size of 0.001 ± 0.004 mm^2^ were observed along the hepatic plates in single fields of view. The cells that formed the granulomas were compact. Hepatocytes surrounding granulomas had a bright nucleus with clear nucleoli, and signs of dystrophy were detected in the cytoplasm. None of the histological markers associated with the late stages of apoptosis and cell necrosis were observed (Figure 11).

##### Lungs

**Figure 12:**
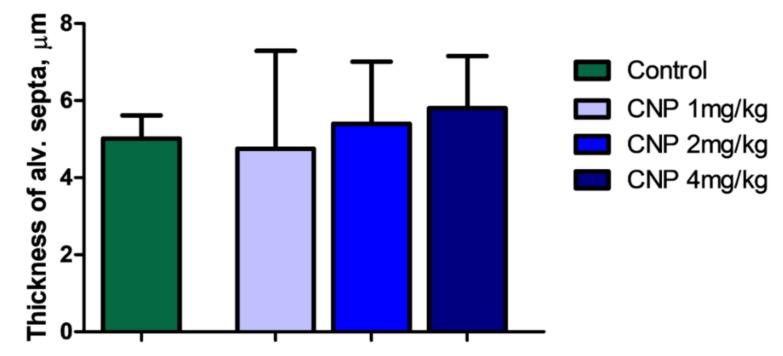
Thickness (μm) of rat alveolar septa at 14 days after intravenous administration of CNP suspension. Mann–Whitney test. Median ± range.

The alveolar septa in the pulmonary interstitium of control animals contained a polymorphic cell pool consisting mainly of the components of the macrophage and lymphoid series as well as eosinophilic leukocytes. Single alveolar macrophages were observed in the alveoli. The CNP administration caused no changes in the thickness of the alveolar septa (Figure 12). However, a marked increase in the number of macrophages and lymphocytes occurred on the 14th day of observation. This is a characteristic response at this time, and the representation of eosinophils, mast cells, and alveolar macrophages did not significantly change. One feature indicative of histological changes in the lung tissue, as well as in the liver, were single sites of granulomas, which were manifestations of the response of the resident pool of macrophages (Figure 12).

#### 3.3.4. Results of Immunohistochemical Analysis

**Figure 13:**
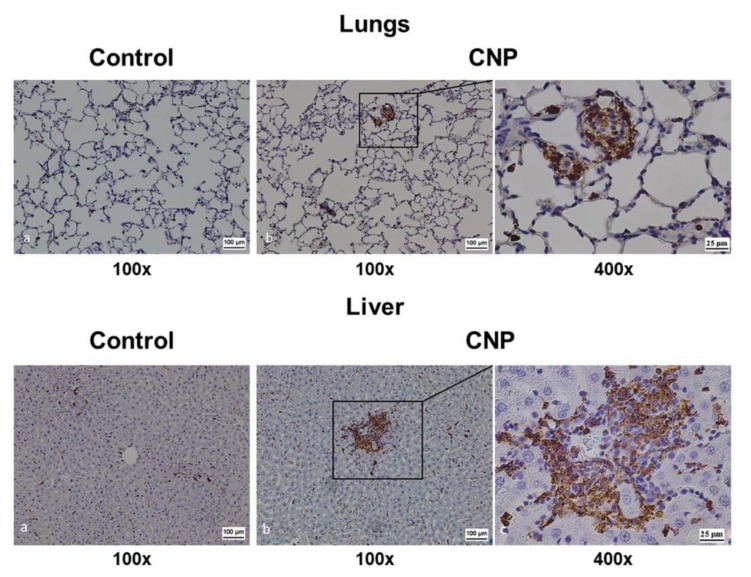
The lungs and liver of rats at 14 days after CNP administration 4 mg/kg. Immunohistochemistry analysis with antibodies to CD68+. Areas with CD68+-macrophage granulomas.

The IHC analysis with antibodies to CD68 revealed that the granulomas found in the lungs and liver at 14 days after intravenous CNP administration were formed by CD68+ macrophages (Figure 13).

#### 3.3.5. Histological Verification of CNP Internalization

**Figure 14:**
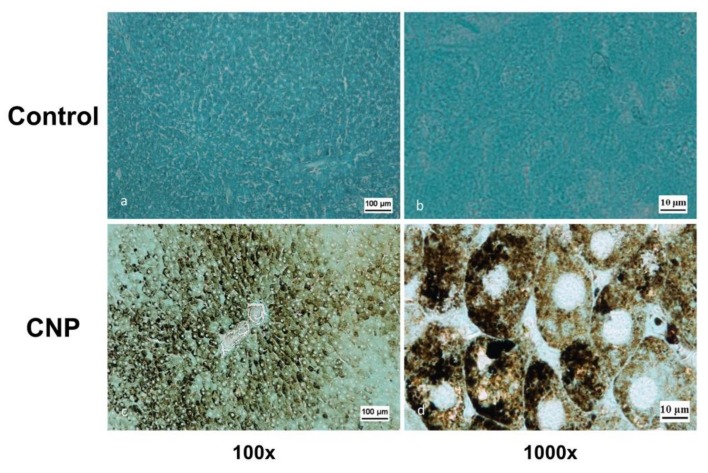
Rat livers 2 h after administration of CNP suspension (16 mg/kg). Grocott’s staining technique: (**a**) hepatocytes of control group (100×), (**b**) the same hepatocytes at magnification 1000×; (**c**) CNP group, hepatic lobule, area of the central vein, hepatocytes with internalized CNPs (100×); (**d**) the same hepatocytes at magnification 1000×.

The maximum staining intensity from Grocott’s technique (black inclusions) was observed in the liver 2 h after the administration of the CNP suspension. Areas of the hepatic lobules with CNP internalization were clearly identified by the distribution of argentophilic inclusions in hepatic lobules (Figure 14c). Stained inclusions were detected in hepatocytes and Kupffer cells. A significant proportion of the cells contained large granules, often filling the entire cytoplasm (Figure 14d).

**Figure 15:**
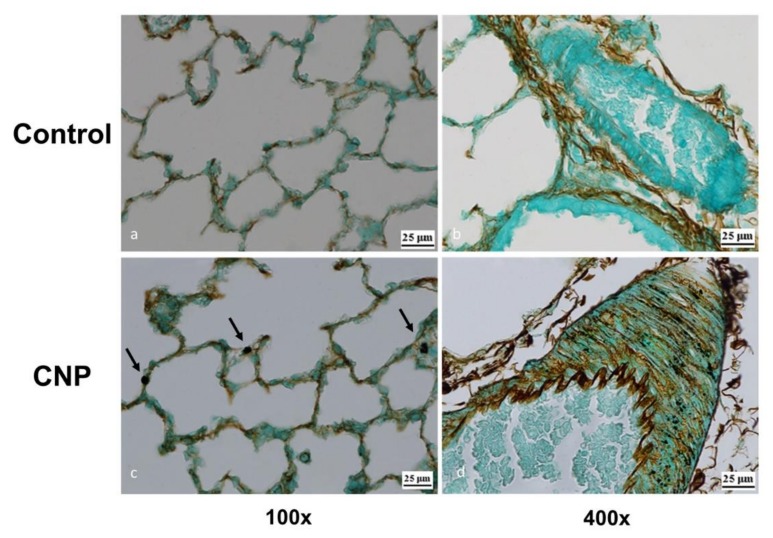
Rat lungs 2 h after administration of CNP suspension (16 mg/kg). Grocott’s staining technique revealed: (**a**) the alveoli of the lung of control rat (400×); (**b**) control rat lung artery (400×); (**c**) interstitial macrophages (arrows) containing CNPs in the alveolar septa (400×); (**d**) rat pulmonary artery and muscle layers with stained CNPs (400×).

The argentophilic substrate was less intense in the lung tissue and was located mainly in interstitial macrophages of the interalveolar septa (Figure 15c). Smaller granules and in relatively smaller amounts were observed in the cytoplasm of the alveolar macrophages at that time. A major feature of lung biodistribution of CNPs stained by the Grocott’s technique was the occurrence of large inclusions in adventitia of the arteries and in the walls of the bronchi and bronchioles (Figure 15d).

## 4. Discussion

Table 3 summarizes the biological safety profile of a sample of CNPs with a size of 100 ± 30 nm, as determined by in vitro tests and after a single intravenous administration to rats.

The CNPs caused a significant decrease in ADP-induced platelet aggregation in platelet-rich plasma and a decrease in the prothrombin index, indicating an anticoagulant effect that prolonged coagulation by the “internal” pathway. Similar findings have also been reported for chitosan nanoparticles with a size of 180 ± 25 nm and a positive ζ-potential (+20.7 ± 0.8 mV) [21]. The nanoparticles in the previous study had been obtained by ion gelation with tripolyphosphate, whereas we generated our CNPs by grinding chitosan and filtering through a 200 μm filter. Our CNPs therefore had no crosslinking elements in their structure. This may have maintained a low positive ζ-potential so that CNP binding to plasma proteins prevented platelets destruction by adhesion but reduces ADP-induced platelet activation. The CNPs had a slight positive ζ-potential, which possibly was even further reduced after contact with blood proteins.

According to the literature, chitosan has a known procoagulant effect [39,40]. However, this response is observed on washed platelets devoid of plasma proteins. The opposite result in our experiment can be explained precisely by the presence of protein in the reaction medium, as the interaction between CNP and proteins in the medium is what precludes the interaction of chitosan with platelets. This is suggested to be an agonist-independent process that blocks collagen-induced platelet aggregation and P-selectin expression on platelet membranes, but not GPIIb-IIIa expression [41].

The liver is a major site of accumulation of chitosan nanoparticles after their intravenous administration [26,42,43]. The effect of chitosan on the viability of hepatocytes has been investigated in a number of studies on the creation of artificial liver, where chitosan served as a framework (nanofiber scaffold) for cultures of hepatocytes. The presence of chitosan fibers in the intercellular space, performing a supporting function, improved the function of hepatocytes [44,45]. Cytotoxic effect of chitosan nanoparticles (333 ± 43 nm and 3.3 ± 0.4 mV at pH 7.4) was evaluated in human liver progenitor cells [46]. The internalized nanoparticles caused a reduction in cell viability and proliferation within the concentration range of 0,01% to 1%. In this study, the highest concentration of CNPs in the MTT test was 0,005%, and it was the maximum possible concentration (in the MTT test), because in the stock suspension it was 0.05%. In the study of biocompatibility of intravenously administrated ionic crosslinked nanoparticles based on chitosan and bovine serum albumin did not reveal hepatic toxicity according to the results of biochemical analysis [30]. The authors studied the cytotoxicity of chitosan nanoparticles on epithelial cell lines in an MTT test that showed low cytotoxicity of chitosan-albumin nanoparticles (20 mg/mL for 80% chitosan content in the nanoparticles).

Along with general toxicity, we are also interested in cardiotoxicity of CNPs. We observed decrease of cell viability only after 72 h of exposition with CNPs. The low CNP cytotoxicity in HL-1 cells can also be similarly explained as a consequence of the small positive charge, as this retards CNP adhesion to the membranes of isolated cells (cultured cardiomyocytes) and subsequent nanoparticle internalization. Differences with control cells appeared after 72 h. Three days in culture without passaging are stressful conditions for HL-1 cell line, as monolayer becomes over confluent during this time. In such stressful conditions cell viability is decreased regardless of the concentration of chitosan nanoparticles, while in normal cell density cells well get on with presence of CNPs. We did not observe dose-depending manner of influence of CNPs on cell viability. This confirms that CNPs is not toxic for cells in all tested concentrations.

The results obtained with the MTT test are consistent with the CNP effects observed in the hemolysis test and ADP-induced platelet aggregation. The protein present in the culture medium, as well as in the blood, is adsorbed onto the CNPs and prevents their direct interaction with cell membranes. This explanation is supported by previous MTT test results by Montero et al. (2019) [30], who showed less toxicity for albumin-coated CNPs that contained more albumin than chitosan (chitosan 20%, albumin 80%). Those micelles also had a low positive ζ-potential (+12 mV). Our results are therefore consistent with the literature indicating that in vitro conditions for hemocompatibility and absence of cytotoxicity are a low positive CNP charge and the ability of the CNPs to interact with plasma proteins.

The existing literature also includes conflicting information on the biosafety of in vivo intravenous administration of CNPs [17]. On the one hand, the impossibility of intravenous administration is indicated due to hemolysis and intravascular thrombosis [47]. On the other hand, information on CNP safety has been published [25,26,30,48]. The ability of chitosan, a polycation, to agglutinate erythrocytes in vitro and to cause hemolysis is explained by the electrostatic interactions with erythrocyte membrane structures such as the glycocalyx, proteins, and phospholipids [22,47]. The CNP biocompatibility is also explained by the fact that, under in vivo conditions, negatively charged plasma proteins are adsorbed onto the surfaces of the nanoparticles, thereby preventing erythrocyte aggregation and hemolysis. In our work, the lack of in vivo hemolysis 1 h after intravenous administration indicates CNP hemocompatibility at our selected doses. Similarly, the stable erythrocyte and hemoglobin levels in the blood 24 h later indicate the absence of any significant hemagglutination.

The process of thrombosis during systemic CNP incorporation is directly dependent on the magnitude of the positive CNP charge. Thus, pronounced agglutination, hemolysis, and intravascular thrombosis develops for CNPs with a large charge and a high concentration [6], while CNPs with a small charge adsorb coagulation factors, mainly fibrinogen, upon contact with blood and cause only a weak inhibition of platelet aggregation [41]. In addition, the charge of CNP and its derivatives depends on pH of the medium and is determined by the concentration of amino groups in polymer molecule [21,22]. Resuspension in saline reduces the CNP charge, thereby allowing intravenous CNP administration without the risk of hemolysis [21] at CNP doses of 4–6 mg/kg [42,49,50].

In our experiment, the systemic biosafety of the injected sample was evaluated by monitoring the rat blood pressure. The formation of blood clots in the veins during thrombin infusion leads to hemodynamic disturbances, which are manifested as a sharp drop in blood pressure [51]. A CNP suspension at a dose of 2 mg/kg did not affect the systemic hemodynamics in anesthetized rats, nor did it cause hemolysis. Our in vitro data were also in agreement, as we saw no platelet or plasma procoagulant activation or erythrocyte hemolysis, and cell viability was maintained according to the MTT test results.

We also studied the biodistribution of injected CNPs using an ICG fluorophore as a label [52,53,54]. The use of free ICG, which has well-known pharmacokinetics, as a control indicated that less than 4% of free ICG remains in the blood plasma 20 min after administration. The circulating ICG is mainly captured by the liver and excreted through the bile formation system [52,55,56]. We observed the same dynamics 30 min after the administration of ICG solution. By contrast, injection of ICG-CNPs resulted in a longer residence time in the liver, with no fluorescence observed in the intestinal projection. Fluorescence tomography revealed that 93% of the ICG-CNPs were captured by the liver, in agreement with literature data showing that the liver and spleen become the main target organs of passive nanoparticle biodistribution [13,16,43,57,58]. Interestingly, a small amount of immobilized ICG (6%) was delayed in the lungs. We attribute this to aggregation of a sludge of nanoparticles in the microvasculature during passage through the lungs, indicating that the lungs may be the first barrier organ to intravenously administered CNPs. This phenomenon has already been described and has actually been proposed as a strategy for targeted delivery to the lungs [58]. The peculiarities of the relationship between the CNP biodistribution in the liver and lungs were fully consistent with the histological pattern of CNP internalization obtained in our samples stained by Grocott’s technique: massive CNP internalization in hepatocytes and trace amounts in lung tissue, mainly in resident (interstitial) macrophages.

The low intensity of the inflammatory response on the 14th day after intravenous administration of CNPs in target organs indicates the internalization of the predominant part of CNPs in hepatocytes (visible when stained by Grocott)—cells that are evolutionarily adapted for the elimination and metabolism of foreign (pathological) material. In turn, the observed low macrophage infiltration in the liver can be interpreted as mechanism of removal of foreign material (particles and their agglomerates) that is outside the hepatocytes (for example, accumulation in the space of Disse). At the same time, the products of chitosan biodegradation, glucosamine and N-acetylglucosamine, are not toxic and do not cause death of "foreign-body cells", which in turn contributes to the formation of triggers of reactive inflammation. At the 14-day observation timepoint, the lungs and liver showed signs of a moderate aseptic reactive process in the form of granulomas from CD 68+ macrophages. The literature has repeatedly noted the formation of “foreign body granulomas” around implanted products based on chitosan [20]. Our study is the first to report that intravenous CNP administration can be accompanied by the formation of granulomas in passive biodistribution organs.

One notable physiological effect after a single intravenous administration of CNPs was a dose-dependent decrease in body weight gain on the first day, with subsequent restoration of weight gain (Figure 7). Clinical examination of animals did not reveal any behavioral signs of distress or pain following CNP administration. The available published data indicate that both single and multiple intravenous administrations of CNPs and their derivatives at doses similar to ours are tolerated satisfactorily by animals without inducing a change in body weight [25,30,48,59,60]. The absence of differences in the clinical and biochemical blood tests between the groups suggests that the cause of weight loss was not a “classic” cytotoxic effect. We attribute the weight drop to a systemic elimination process that is energy-consuming and inhibits natural anabolic processes (physiological growth and weight gain in growing organisms). A similar mechanism leads to weight loss during wound healing and convalescence after infections [61,62].

The introduction of dispersed chitosan-containing solutions was not accompanied by an increase in leukocytosis due to incorporation of the dispersed solution and the onset of biodegradation of the particles. The absence of a leukocyte response to the introduction of nanoparticles is explained by the speed of elimination of the majority of nanoparticles from the circulation by hepatocytes. The hepatocyte uptake reduces the fraction of particles that can be captured by the blood cells and resident macrophages (e.g., Kupffer cells, interstitial macrophages of the lungs) that are capable of triggering an immune response. Previous works involving administration of CNPs, either in large doses or repeatedly, have always reported increased leukocyte levels [19,30,63]. Apparently, in these cases, a longer circulation of nanoparticles occurred in the blood and this stimulated intercellular cooperation and increased the proinflammatory cytokine blood levels. Leukocytosis, as a response to the massive incorporation of particles into the blood, is determined by the proliferation and migration of young cells of the monocytic pool to the target organs [19,20,30] with the aim of CNP elimination.

## 5. Conclusions

In vitro tests and in vivo experiments on rats were conducted to determine the toxicity and biodistribution of suspensions of nanoparticles made from low molecular weight chitosan (M_η_ = 138 kDa, DDA – 84%). The CNPs, which had a size of about 100 nm and a slightly positive ζ-potential, showed little antiplatelet and anticoagulant activity and low cytotoxicity to cultured cardiomyocytes when supplied at doses of 1–4 mg/kg. Intravenous CNP administration did not disturb systemic hemodynamics or cause intravascular hemolysis. At 30 min after intravenous administration, CNPs had accumulated mainly in the liver, with predominant internalization in the cytoplasm of hepatocytes, although a small number of CNPs were delayed in the lungs within interstitial macrophages. Observation over 14 days did not reveal any changes in hematological and biochemical parameters of the blood. The absence of weight gain in young (growing) rats on the first day of observation indicated the presence of a systemic elimination response of the body aimed at limiting systemic circulation and at elimination of foreign particles. Reactive changes in the lungs and liver in the form of single granulomas reflected a physiological response of organ cells to a slowdown in the natural elimination of foreign objects in places of passive biodistribution. The absence of leukocytosis, biochemical markers of organ cytolysis, and necrosis and apoptosis patterns in the biodistribution organs at the 14th day of observation indicates that CNPs and their biodegradation products have good biosafety. The findings indicated that CNPs (100 ± 30 nm) with a small positive ζ-potential (+10 ± 1 mV) are relatively well tolerated by rats when administered intravenously. The results obtained here will now allow us to continue this research on the biological safety of CNPs in a chronic experiment.

## Figures and Tables

**Table 1:** Indicators of coagulation hemostasis following addition of chitosan nanoparticles to human plasma. Mean ± standard error. n/s – non-significant data. * – *p* < 0.05 compared to control.

Parameter	Control *n* = 5	CNP 0.25% *n* = 5	*p*
APTT, s N 28–40	34.8 ± 1.1	31.7 ± 1.2	0.09 n/s
PI, % N 85–115%	99.2 ± 1.7	60.5 ± 1.8 *	0.005
TT, s N 14–20	16.4 ± 1.7	20.3 ± 1.5	0.051 n/s

**Table 2:** Indicators of systemic hemodynamics following administration of a CNP suspension.

	Saline		CNP 2 mg/kg	
	MAP, mm Hg	HR, bpm	MAP, mm Hg	HR, bpm
Baseline	136 [134–148]	396 [372–420]	121 [105–142]	396 [366–408]
Beginning of administration	136 [130–151]	396 [365–420]	134 [110–146]	384 [361–402]
End of administration	139 [126–147]	396 [366–420]	138 [115–145]	395 [364–402]
5 min after administration	126 [117–148]	384 [344–420]	143 [120–144] *p = 0.026	396 [371–401]
30 min after administration	134 [110–146]	384 [361–402]	136 [118–141]	396 [383–413]
60 min after administration	–	–	124 [104–137]	408 [354–417]

*—*p* compared to baseline (paired Wilcoxon test). Median and the 25th and 75th percentiles (Me [25–75%]).

**Table 3:** Biological safety parameters of nanosized (100 nm) CNPs.

Assessment Parameter		Effect of CNP Suspension
Platelet adhesion		Decrease
Platelet aggregation		Decrease
Coagulation potential		Decrease
Hemolytic activity		No
Cultural cytotoxicity		Low
Influence on systemic AP		No
Decrease in body weight gain		Yes
Biodistribution	Liver	93%
	Lungs	6%
Intensity of histological reactive changes (granuloma formation)	Liver	Low
	Lungs	Low
